# Severe pneumonia in adults caused by *Tropheryma whipplei* and *Candida* sp. infection: a 2019 case series

**DOI:** 10.1186/s12890-020-01384-4

**Published:** 2021-01-15

**Authors:** Wei Li, Qun Zhang, Yanling Xu, Xiyue Zhang, Qian Huang, Zhenzhong Su

**Affiliations:** 1 Department of Respiratory and Critical Care Medicine, The Second Hospital of Jilin University, Changchun, Jilin China; 2 Department of Radiology, The Second Hospital of Jilin University, Changchun, Jilin China

**Keywords:** *Tropheryma whipplei*, *Candida*, Pneumonia, Metagenomics next-generation sequencing, Bronchoalveolar lavage

## Abstract

**Background:**

Whipple’s disease is a chronic infectious disease caused by the Gram-positive bacterium *Tropheryma whipplei* (TW), which not only affects the gastrointestinal tract and causes malabsorption of nutrients, but several other systems, such as the cardiovascular system, central nervous system, the joints, and the vascular system, can also be simultaneously involved. The aim of this report was to be able to alert the clinician to severe pneumonia caused by TW combined with *Candida* sp.

**Case presentation:**

The case study was conducted on patients in September and November 2019. After routine examination and treatment, the results were not satisfactory. A bronchoalveolar lavage (BAL) using metagenomics next-generation sequencing was conducted on two adults who presented with fever, cough, and progressive dyspnea and who had no history of gastrointestinal symptoms, immunodeficiency diseases, or use of immunosuppressive agents. TW and *Candida* sp. were detected in in BAL.

**Conclusions:**

This is a report of life-threatening pneumonia caused by TW combined with *Candida* sp. in a Chinese population.

## Background

*Tropheryma whipplei* (TW) is a Gram-positive bacterium originally named in 1907 after George H. Whipple as Whipple’s disease (WD) [1]. WD is a chronic and rare systemic infectious disease affecting the gastrointestinal tract and causing arthritis and weight loss[2]. The 16S ribosomal TW DNA was first identified in 1991 from a small intestinal biopsy of typical WD using molecular assays for nucleotide sequencing and amplification [3]. The common symptoms are fever, abdominal pain, diarrhea, weight loss, and joint pain. Classic diagnosis is made through a histological analysis of a small-bowel biopsy. TW was first isolated and cultured in 2000 from the heart valve of a patient with endocarditis [4]; subsequently, the bacteria were also cultured from other tissues or bodily fluids from WD patients, including the duodenum, feces, cerebrospinal fluid, lymph nodes, skeletal muscle, skin, and joint fluid [5]. As health-care professionals learned more about this bacterium, they found that TW does not cause only WD, a rare condition, but also chronic infections [6], such as endocarditis, nervous system infections, uveitis, arthritis and joint infections, and simple adenosis [2, 7], and acute infections, such as acute gastroenteritis [2], travelers’ diarrhea [8], and pneumonia [9].

In this case study, we present two adult patients who were admitted to the Department of Respiratory and Critical Care Medicine at the Second Hospital of Jilin University with fever, dry cough, and dyspnea, which was subsequently diagnosed as TW pneumonia along with a *Candida* sp. infection using metagenomics next-generation sequencing (mNGS) analysis of the bronchoalveolar lavage (BAL) fluid. One patient died from the infections, which emphasizes that they can be lethal without early diagnosis and treatment.

## Case presentation

### Case 1

In September 11, 2019, a 39-year-old woman was hospitalized for coughing, difficulty breathing, and a low-grade fever (38.0 °C) that persisted for 25 days, and a persistent sputum discharge 3 days before admission. During the course of the disease, there were no other symptoms (e.g., dizziness, headache, nausea, vomiting, anorexia, fatigue, abdominal pain, diarrhea, or weight loss). Her medical records included two cesarean sections but no other history of illness, and she was not taking any hormones or immunosuppressive agents. The patient did have a history of working for months in an unventilated office.

#### Diagnoses

**Fig. 1:**
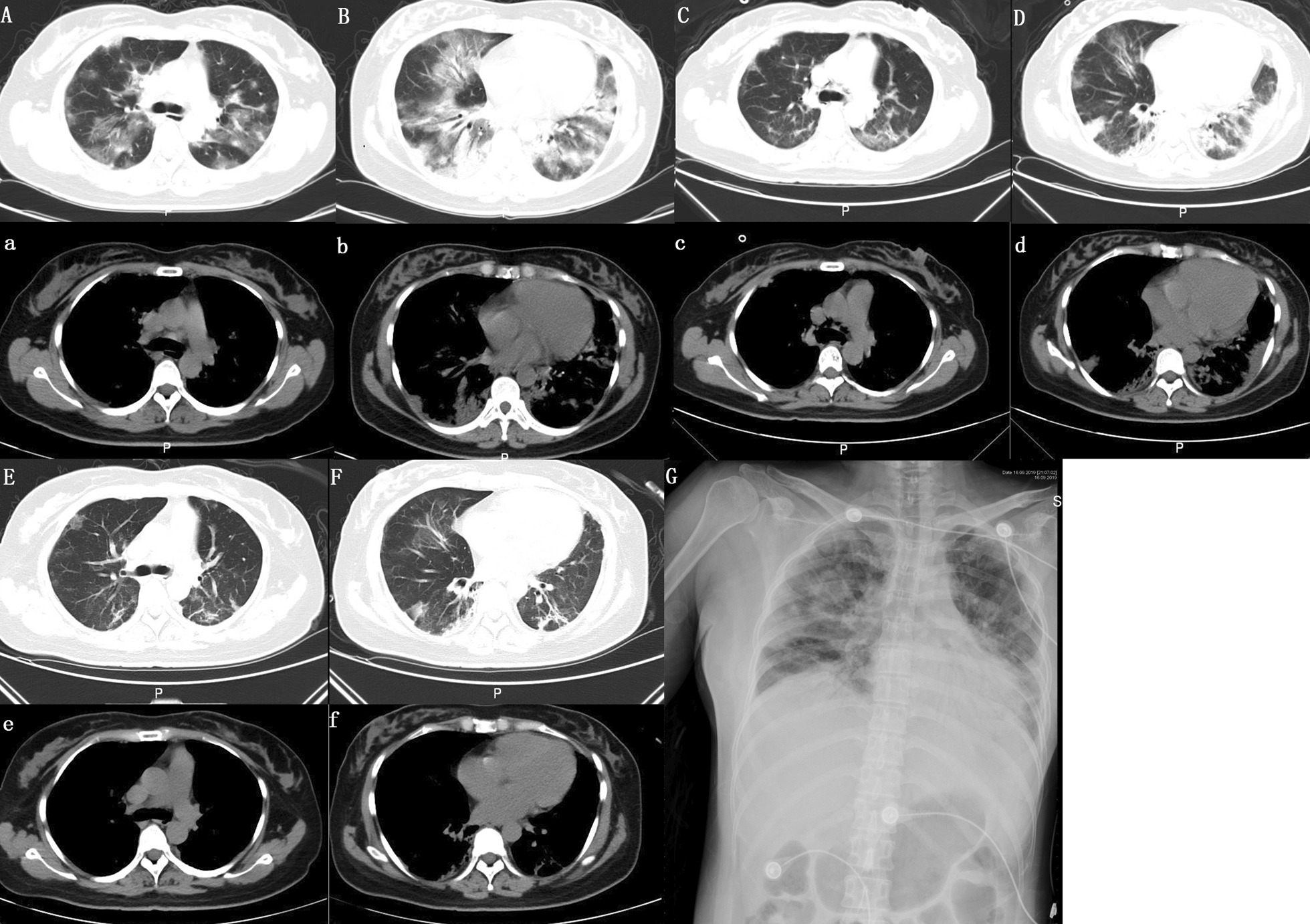
Computed tomography (CT)/X-ray images of the chest of patient 1. Uppercase letters indicate lung windows; lowercase letters indicate mediastinum. **A**, **B**, **a**, **b** Lung CT on the day of admission; **G** bedside chest X-ray at 5 days; **C**, **D**, **c**, **d** lung CT at 13 days; **E**, **F**, **e**, **f** lung CT at 17 days

On admission to the hospital, the patient’s body temperature was 38.1 °C. In addition, the patient had developed symptoms of shortness of breath (35 bpm) and severe hypoxemia (PaO_2_, 62 mmHg; FiO_2_, 0.80). During auscultation, the patient had good air sounds in both lungs and medium moist rales. Hematological results showed normal white blood cell counts (6.6 × 10^9^/L), while neutrophil counts were slightly higher, accounting for 78.7% granulocytes (or neutrophils) (Additional file 1: Fig. 1); C-reactive protein at 102 mg/L was 13.7 times higher than normal; and there were fibrinogen in the plasma. The proteinogen level was 4.46 g/L. There were a large number of Gram-positive bacteria in the sputum smear, but no bacterial growth was observed in the culture. Procalcitonin and fungal d-glucan levels were normal. The respiratory virus test was negative. Computed tomography of the lungs showed multiple bilateral pneumonia (Fig. 1A, a, B, b).

#### Interventions and outcome

The patient’s respiration was immediately monitored and supplemental oxygen was given to improve low oxygen saturation, and antibiotics (moxifloxacin and piperacillin sodium sulbactam) and antifungal therapy (micafungin) were administered together with glucocorticoids. The dosage of glucocorticoids was gradually reduced. After 5 days of treatment, the patient’s condition worsened, and a bedside chest X-ray revealed that the disease had progressed (Fig. 1G); The mNGS analysis of the BAL fluid revealed TW and *Klebsiella pneumoniae*, with the number of sequences of the former being dozens of times higher than that of the latter, along with a considerable number of sequences of *C. albicans*. Given that the broad-spectrum antibiotics had no effect and that the bacteria culture was negative, we considered using antibiotics specifically against TW; therefore, we adjusted the treatment protocol to include a combination of sulfamethoxazole tablets and meropenem and antifungal therapy (fluconazole). After 17 days, computed tomography of the lung revealed that the lesions were significantly absorbed (Fig. 1E, e, F, f).

### Case 2

On October 31, 2019, an 81-year-old man was admitted to the hospital for coughing and difficulty breathing for 15 days. No fever, joint pain, abdominal pain, diarrhea, or other symptoms were observed. The patient’s medical records included coronary stenting and hypertension. There were no autoimmune or immunodeficiency diseases. No immunosuppressive agents were used but he had regularly taken hot spring baths.

#### Diagnoses

**Fig. 2:**
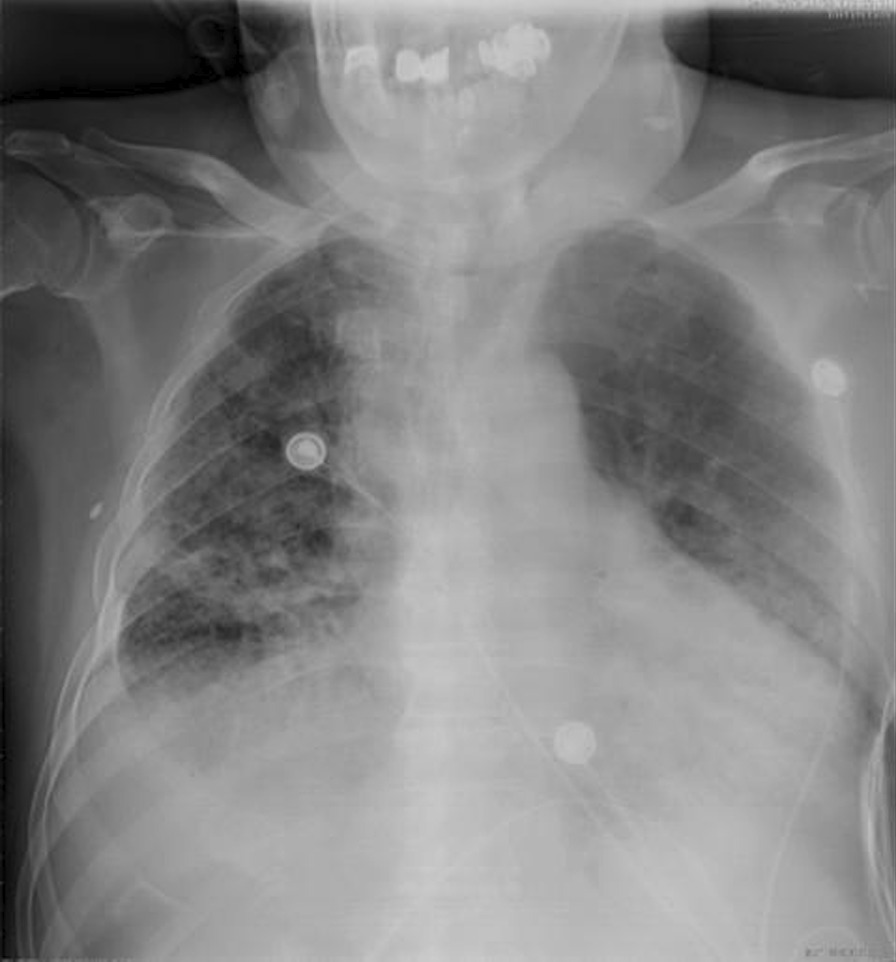
Bedside chest X-ray. Multiple lungs with patchy high-density shadows

On admission to the hospital, the patient’s body temperature was 36.7 °C. In addition, he developed symptoms of shortness of breath (30 bpm) and severe hypoxemia (PaO_2_, 44 mmHg; FiO_2_, 0.80). During auscultation, the patient’s breath sounds weakened, and rales were heard in both lower lungs. Blood tests showed a normal white blood cell count (8.1 × 10^9^/L), 68.9% neutrophils, an increase in C-reactive protein to 116 mg/L (15.6-fold higher than normal), and an increase in plasma fibrinogen levels to 4.75 g/L. X-rays of the lungs showed bilateral pneumonia (Fig. 2). Respiratory virus (influenza A, influenza B, parainfluenza 1,2,3, respiratory syncytial, cytomegalovirus, rubella, adenovirus, rhinovirus, coxsackie) IgM antibody test results were negative, and procalcitonin and fungal d-glucan levels were normal. The mNGS analysis of the BAL fluid revealed only TW, and *C. glabrata* and *C. tropicalis*.

#### Interventions and outcome

The patient’s respiration was immediately monitored and oxygen supplementation was provided to improve hypoxia. Antibiotics (tigecycline and piperacillin sodium sulbactam) and antifungal therapy (voriconazole) were prescribed. The dosage of glucocorticoid (methylprednisolone) was 80 mg/day. After 3 days of treatment, the patient’s condition worsened and he was transferred to the intensive care unit (ICU). The mNGS results showed TW and *Candida* sp. infections; therefore, the treatment was adjusted to include meropenem plus compound sulfamethoxazole tablets and voriconazole. The dosage of glucocorticoid (methylprednisolone) was increased to 160 mg/day. The patient’s condition continued to deteriorate rapidly and oxygenation continued to decline; however he refused extracorporeal membrane oxygenation and died 5 days after admission.

## Discussion and conclusions

Types of TW infections reported in recent years have increased. In acute infections, acute gastroenteritis is most common in some impoverished countries, such as Senegal and Ghana; however, in economically advanced countries, such as the United States and France, there are also reports of bacteria isolated from BAL fluid in children and adults with pneumonia, especially adults in ICU [10, 11]. In our case report, we describe two patients in China with severe pneumonia who had coinfections of TW and *Candida* sp. detected from BAL fluid using mNGS.

Some scholars have observed that TW causes acute pneumonia (Table 1). Harris et al. [12] have reported the detection of *TW* in a child with interstitial lung disease, which may be a type of pneumonia with a special pathogen infection caused by TW, by rRNA sequencing. Bousbia et al. [9] have detected TW gene sequences in 6 (3%) of 210 BAL samples using 16S rDNA and specific quantitative polymerase chain reaction (PCR) and found that TW was the only pathogen in some immunocompromised patients with pneumonia who were admitted to ICU. Lozupone et al. [13] have found that in the BAL from HIV-infected patients, TW has a higher prevalence and relative abundance than that in HIV-negative subjects. Studies have shown that TW may also be present in the saliva of asymptomatic individuals [14] and that this can inhaled from the oral flora, resulting in pneumonia. In one of the two patients we studied, TW was the only bacteria identified. Fenollar et al., have suggested that TW is the cause of community-acquired pneumonia [10], while Bousbia et al., have suggested that TW causes both acquired and aspiration pneumonia after inhaling bacteria present in the patient’s saliva [9]. Another large-sample study has shown that TW infection has nothing to do with the patient’s immune status [15].

**Table 1:** Studies of *Tropheryma whipplei* detection in infectious diseases of the lung

Samples	Method	Comments	References
BAL and mouths of 82 HIV-positive and 77 HIV-negative subjects	qPCR	Higher prevalence and relative abundance of TW in BAL in HIV-positive individuals	[13]
BAL and induced sputum samples in 76 HIV-infected participants	PCR and sequencing	Frequency of TW in either BAL or IS was 43.4%	[22]
bronchial biopsy specimens of a man with a history of intermittent fever and arthritis	Immunoreactivity and periodic acid-Schiff	The pulmonary symptoms preceded the development of gastrointestinal manifestations	[7]
BAL fluid samples representing suspected or confirmed pneumonia	PCR	TW was detected in 6 of 210 BAL fluid samples	[9]
BAL fluid of a patient with diffuse pulmonary parenchymal micronodules	Culture and qPCR	Isolated TW and confirmed its role as a respiratory pathogen	[10]
BAL fluid of a patient with pneumonia and active HIV-2 infection	PCR	TW be considered in the differential diagnosis of pneumonia in patients with advanced HIV infection	[23]
BAL fluid of patients with TW in BALs and controls	PCR	No difference was observed regarding immunocompromised status. This study adds evidence for a causative role of TW in pneumonia	[15]

BAL, bronchoalveolar lavage; PCR, polymerase chain reaction; qPCR, quantitative PCR; TW, *Tropheryma whipplei*; HIV, human immunodeficiency virus; IS, induced sputum

In the present cases, the patients were admitted to our hospital with symptoms of fever, cough, expectoration, and dyspnea, but had no known immunocompromising conditions, such as history of taking glucocorticoids or organ transplantation; however, the working or living environment of these two people is a relatively confined space. Laboratory tests revealed elevated inflammation indicators, implying a possible bacterial infection. The mNGS results from the BAL fluid showed that the pathogens most possibly were TW and *Candida* sp. Interpretating the results of bronchoalveolar lavage fluid mNGS must be combined with the patient’s clinical manifestations, previous treatment procedures, sampling methods, and efficacy of targeted anti-infective treatments. Certain special identifications are also sometimes required. As TW infections were not previously diagnosed in our hospital, TW special detection methods such as 16S PCR, TW-specific PCR, or TW-specific culture were not available. Thus, despite both patients being critically ill, neither had a pathologically confirmed TW infection. However, since TW infections combined with severe acute pneumonia were quite rarely detected in the area as in the literature, the TW infection was diagnosed clinically. The successful special treatment against TW in case 1 also supports the diagnosis.

Therefore, we adjusted the treatment against that pathogen. Ruben AV Dolmans [16] reported that many drugs are used to treat TW infection, including penicillin, streptomycin, tetracycline, ceftriaxone, meropenem, compound trimethoprim, doxycycline, and hydroxychloroquine. According to a randomized controlled study, 40 patients were successfully treated with ceftriax-one (one 2-g dose/day) or meropenem (three 1-g doses/day) for 14 days followed by oral co-trimoxazole (combination trimethoprim and sulfonamide) for 12 months. In patients who are intolerant to ceftriaxone, meropenem can be an alternative, while for patients who are intolerant to co-trimoxazole, doxycycline can be used. We applied meropenem plus compound trimethoprim. For case 1, we first applied piperacillin and sulbactam combined with moxifloxacin, but the effect was not good. The antibiotics were replaced with meropenem and compound trimethoprim. In case 2, tigecycline combined with piperacillin and sulbactam was used as the initial treatment. With reference to the treatment experience of case 1, we also used meropenem and compound trimethoprim. We adjusted the treatment from compound sulfamethoxazole tablets and voriconazole to a combination of meropenem, sulfamethoxazole tablets, and voriconazole.

One patient’s prognosis was favorable, while the other patient died from severe infection and poor oxygenation after refusing respiratory support treatments. WD is usually related to innate immune activation defects [13]; However Lagier et al. [2] have found that sewer workers, homeless people in shelters, healthy individuals in rural areas, and families of patients had higher TW detection rates. Our patients were also healthy individuals within a specific working or living environment, such as an unventilated office or frequent exposure to a hot spring. Combined with previous researches, it is hypothesized that the specific environment increases the chance of TW infection in a healthy individual.

Sulis et al. [17] have reported a case of TW in which no specific risk factors for opportunistic infections were identified in patients with candidal esophagitis except TW infection; therefore, they hypothesized that the candidal infection was directly related to WD. Moussawi et al. [18] have proved using a mouse model that TW alone cannot invade tissues but can smolder in cells, including macrophages, by inhibiting the xenogenic phagocytosis process, a selective autophagy that targets pathogens. Some macrophages appear to play a role in limiting the invasion of fungi on mucosal surfaces [19]; Therefore, the peripheral flagellates and *Candida* sp. may work synergistically to cause severe infections and even fatal pneumonia.

Unfortunately, the two patients reported were severely ill, had poor oxygenation, and failed to undergo a pathological examination. TW is an intracellular pathogen that requires cell culture medium, harsh culture conditions, and extended culture time [10, 20]. In lung diseases, PCR is often used to detect TW from BAL fluid (Table 1). Our study showed that mNGS could efficiently screening TW and coinfection pathogens. Harris et al. [21] have suggested that detection of unexpected bacteria using rRNA sequencing may explain the failure to respond to standard treatment in children with cystic fibrosis. Our method using mNGS may be more clinically advantageous in providing a broader perspective on airway bacterial infection than doing routine bacteria cultures and, thus, can screening targets and quickly alert the clinician to further clinical evaluation in severe pneumonia, especially for some hospitals that do not have sufficient pathogen detecting conditions; however, it remains unclear whether there is a close relationship between TW and *Candida* sp. in pneumonia and whether mNGS can achieve the same results as PCR.

Whipple pneumonia is a disease relatively difficult to diagnose, and it easily misdiagnosed or missed altogether. Doctors should be wary of the disease in the patients who are immunocompromised, are exposed to a specific environment, or have a history of contact with TW patients with pneumonia. Perhaps the mNGS test using BAL is a better diagnostic method, which can simultaneously identify WD and co-infections such as *Candida*. The disease has a high fatality rate, and early diagnosis and early treatment are recommended.

## Supplementary information

**Additional file 1.** Supplemental Fig. 1. Indicators of infection and glucocorticoid changes in case 1. (**A**) White blood cell count and percentage change in blood routine; (**B**) Procalcitonin changes; (**C**) Glucocorticoid changes.

## Data Availability

All data generated or analyzed during this study are included in this published article.

## Abbreviations

WD: Whipple’s disease
TW: *Tropheryma whipplei*
BAL: bronchoalveolar lavage
mNGS: metagenomics next-generation sequencing
PCR: polymerase chain reaction
QPCR: quantitative PCR
HIV: human immunodeficiency virus
ICU: intensive care unit
